# Quantifying the Number of Discriminable Coincident Dendritic Input Patterns through Dendritic Tree Morphology

**DOI:** 10.1038/srep11543

**Published:** 2015-06-23

**Authors:** Antonio G. Zippo, Gabriele E. M. Biella

**Affiliations:** 1 Institute of Biomedical Imaging and Physiology, Department of Biomedical Sciences, Consiglio Nazionale delle Ricerche, Segrate (Milan), Italy

## Abstract

Current developments in neuronal physiology are unveiling novel roles for dendrites. Experiments have shown mechanisms of non-linear synaptic NMDA dependent activations, able to discriminate input patterns through the waveforms of the excitatory postsynaptic potentials. Contextually, the synaptic clustering of inputs is the principal cellular strategy to separate groups of common correlated inputs. Dendritic branches appear to work as independent discriminating units of inputs potentially reflecting an extraordinary repertoire of pattern memories. However, it is unclear how these observations could impact our comprehension of the structural correlates of memory at the cellular level. This work investigates the discrimination capabilities of neurons through computational biophysical models to extract a predicting law for the dendritic input discrimination capability (M). By this rule we compared neurons from a neuron reconstruction repository (neuromorpho.org). Comparisons showed that primate neurons were not supported by an equivalent M preeminence and that M is not uniformly distributed among neuron types. Remarkably, neocortical neurons had substantially less memory capacity in comparison to those from non-cortical regions. In conclusion, the proposed rule predicts the inherent neuronal spatial memory gathering potentially relevant anatomical and evolutionary considerations about the brain cytoarchitecture.

Neurites are important neuron compartments that distinctly characterize the cytoarchitecture of nervous tissues and realize intercellular communications. Specifically, dendrites are complex tree shaped structures that take part in neurotransmission through specialized membrane protrusions (spines) which represent the preferential sites for the neurotransmitter reception. Remarkably, dendritic spines and trees are considered part of the morphological correlates of structural plasticity and causal modifications of the dendritic tree morphology (synaptogenesis, spinogenesis and branch remodeling) have been related to learning^1^,^2^. Hence, the dendritic tree inherently represents an attractive perspective to study structural learning and long-term memory at the cellular level^3^,^4^,^5^,^6^.

From a functional point of view, dendrites were generally recognized as passive electrotonic compartments, which conveyed and integrated the electrical field variations triggered by ionic channel openings at the post-synaptic terminals. However, recent studies highlighted that in dendritic trees, a rich repertory of ionic channel mechanisms modulate incoming and back-propagated running signals (dendritic spikes) by local voltage dependent ionic channels^7^. Indeed, a prominent work reported that mechanisms of non-linear synaptic N-Methyl-D-aspartic acid (NMDA) dependent activations have been shown to likely discriminate input patterns along the branches of dendritic trees. The authors argued that “pyramidal cell dendrites can act as processing compartments for the detection of synaptic sequences”^6^,^8^,^9^, a tangible property observable in the waveforms of the excitatory post-synaptic potentials (EPSPs). Furthermore, by means of biophysical models, other authors showed that neurons with larger dendritic trees have greater computational power^10^,^11^,^12^, however without supplying a quantitative analysis. In such a perspective, dendritic branches acting as computational blocks for neural information processing could potentially sustain the significant computational loads, currently missing in present analytic perspectives. In the last decades, many works focused on the electrodynamical properties of the dendritic tree nonetheless, it is not yet clear how morphological features of dendritic trees are related to or may sustain their functional counterparts.

Complementarily, a recent line of research showed that functionally relevant synaptic inputs, resulting in strongly correlated inputs, are organized in clusters of synapses within dendritic branches thus promoting robust propagations of large dendritic depolarizations^13^,^14^,^15^. These evidences came from several experimental setups (including *in vivo*) and have been observed in many brain regions, generally called *synaptic clustering*^6^,^16^. Therefore the synaptic clustering hypothesis provides a spatial constraint for correlated input intensely restricting the theoretical number of possible input configurations along dendrites.

In this work we repropose the idea that dendritic trees are not simple input integrators but, well more broadly, rather recognizers of input patterns and that such recognition takes place in each dendritic branch. This work has two main scopes: the first is to quantitatively assess the impact of these novel facts about dendrites in terms of number of recognizable input patterns per neuron. The second aim is to evaluate the functional consequences generated by the resulting quantitative relationship within the current neuroanatomical data.

In our computational framework, neuron models are composed of two parts: the specification of the cell geometry and the definition of the biophysical properties. Since, such properties comprise many fundamental parameters that can strongly affect the results and most of them are inaccessible, we designed an optimization strategy, based on genetic algorithms, that maximized the number of discriminable input patterns by exploring a multidimensional parameter space composed of five variables: the spine density, the spine spatial distribution, the membrane resting potential, the NMDA and AMPA receptor concentrations.

In a recent study, Cuntz *et al* proposed a scaling law relating the total dendritic length, the number of branching points and synapses^17^,^18^. By exploiting such law, the putative number of spines for each dendritic branch can be extracted to infer the spine distribution along the dendrite segments. Since the Cuntz law has not had an exhaustive experimental support, we further investigated different values of synaptic density to address possible effects. The spatial distribution of synapses represented an additional open question because it is still debated whether dendritic spines are placed according to deterministic schemes (e.g. the 3D helix-shaped Purkinje cells) or to random arrangements^19^,^20^,^21^,^22^. Eventually we included other biophysical properties such as the membrane resting potential and the number of NMDA and *α*-Amino-3-hydroxy-5-methyl-4-isoxazolepropionic acid (AMPA) receptors because they could drive relevant consequences on the input discriminability.

To target the first aim, we quantified the number of discriminable patterns in relation to two relevant morphological properties: the number of dendritic branches and the total dendritic length. In order to address the second aim instead we retrieved the morphological data from the largest open repository of neuron reconstructions (neuromorpho.org). We provided a set of potential inferences by performing comparisons across neuron types, animal species and brain regions suggesting both new perspectives and roles of dendritic morphological features within the mainstream of animal species and of brain region phylogeny.

## Results

**Figure 1:**
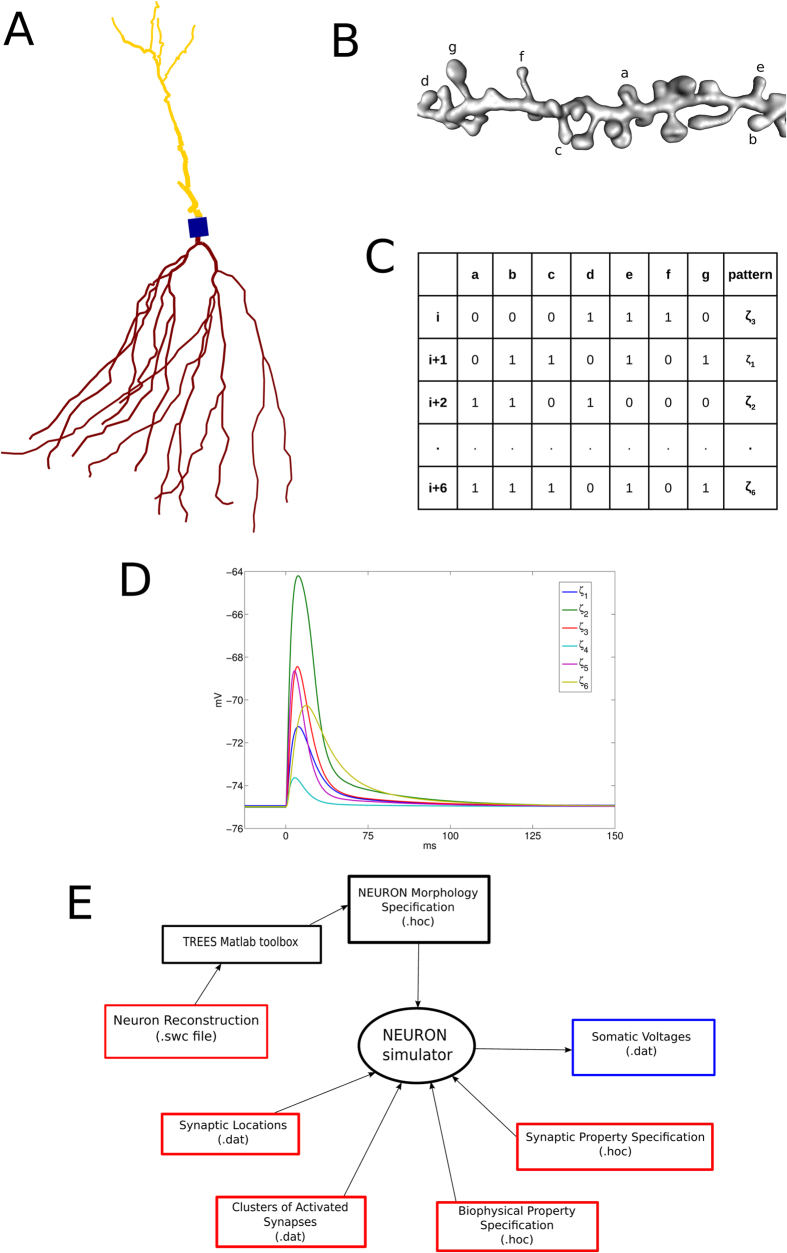
The computational approach proposed in the work. (**A**) A representation of a reconstructed neuron. (**B**) 3D representation of dendritic segment where we arbitrary labeled seven spines. (**C**) Different patterns 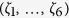 of simultaneous activation for the seven spines that correspond to the six different waveforms in (**D**). (**E**) A scheme of the computational framework where boxes in red represent the variable input files, boxes in black represent constant input files (whether they connect the central ellipse) and the blue box represents the only output file. Reconstructed neurons are first converted in the NEURON neuron geometry syntax, then once specified the synaptic positions along the dendritic tree and which synapses will be active, the NEURON simulation produces a set of somatic voltages that will be analyzed by the algorithm 0 to quantify how much waveforms are distinguishable.

**Figure 2:**
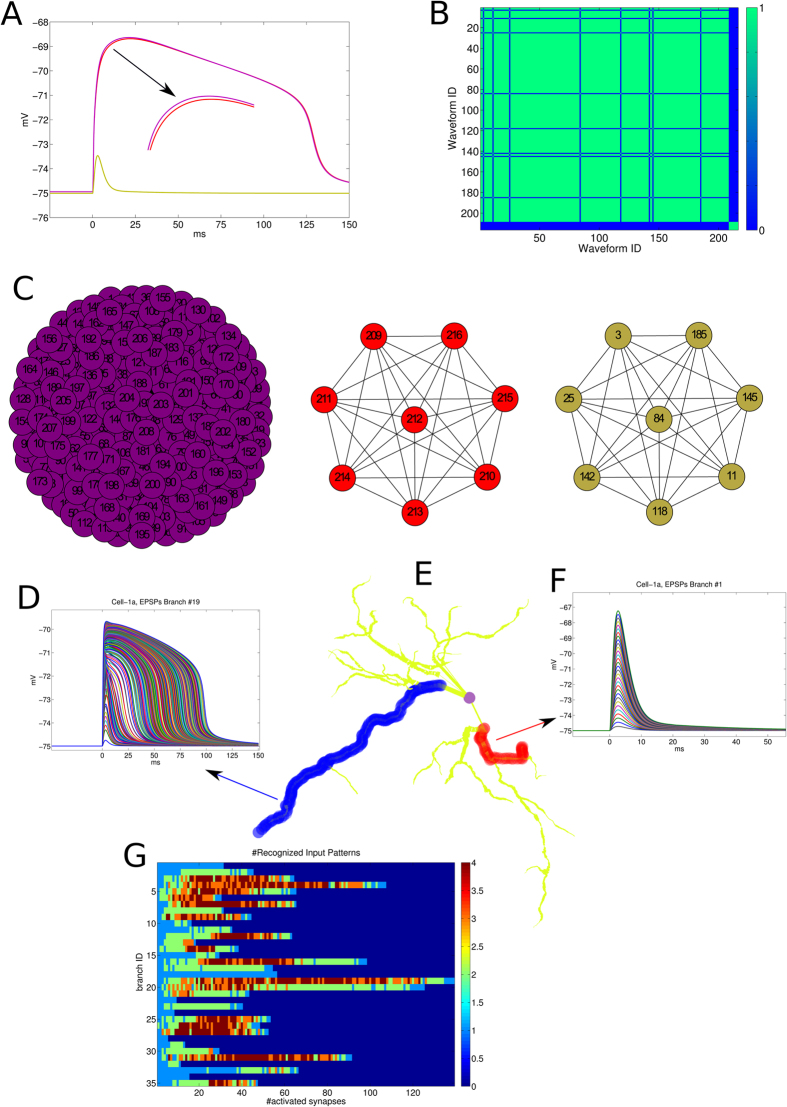
Explanation of the method devised to quantify the waveforms discriminability. In this toy example are used 208 activation patterns along a fixed dendritic branch. (**A**) The 208 somatic waveforms can qualitatively be grouped in three groups (yellow, red and purple). (**B**) The method first computes a similarity matrix which can be seen as the adjacency matrix of a graph. (**C**) The number of connected components, i.e. the number of complete disjoint graphs corresponds to the number of previously visually identified discriminable waveforms (purple, red, yellow). (**D**,**F**) All waveforms discrimable respectively by the branches highlighted in blue and red of the dendritic tree of the cell (Cell-1a, Mouse, Ventral Thalamus) displayed in (**E**). The soma centroid is highlighted in purple. (**G**) The result of the discriminable analysis for all dendritic branches of the cell is shown.

In this work we planned to quantify the discriminability of dendritic correlated and spatially clustered inputs starting from some morphological features of dendritic trees. We used a purely computational approach based on the NEURON simulator and on the large repository of neuron reconstructions, neuromorpho.org (Fig. 1F). Primarily, we wondered if a general rule linking discriminability capacity and morphological features could be extracted and, subsequently, we adopted such a rule for a comparative neuroanatomical inspection spanning species, neuron types and central nervous system regions. As a rule, two dendritic input patterns are considered as discriminable according to a simple criterion which establishes whether a relevant number of data points (equivalent to 10 ms, see Section 0 for further details and Fig. 2A–C) differs between two somatic waveforms.

### Input Discriminability

We developed a computational framework to investigate the morphological correlates of input discriminability in dendrites. To this aim, we designed an ordinary genetic algorithm to tune up a set of biophysical properties to be combined to geometries obtained from reconstructed neurons. These included the density of AMPA and NMDA receptors, the synaptic density, the spine spatial distribution and the membrane resting potential. Indeed, the number of AMPA and NMDA receptors has been shown to be critical for the input discrimination as well as the resting potential^8^. Furthermore, because it is generally unknown how spines are located along dendrites^19^,^20^,^21^,^22^, we considered two models to profile the spatial distribution of spines along the dendrites, i.e. the equidistant and the uniformly random. In the first, the spine distance is constant and inversely proportional to the spine density while in the second model the spine location was extracted by means of a random uniform distribution. At last, to establish the spine density we exploited the Cuntz’s law which relates the total dendritic length, the volume and the number of synapses. We also evaluated the potential effects on results assuming different values. The estimation of the discriminability capacity of cell (M) involved hard computations that can last several weeks and this issue limited a broad analysis of the entire neuromorpho dataset which contains more than ten thousand neuronal reconstructions. For this reason, we selected a sample of 100 neurons randomly chosen from the entire neuromorpho dataset. A sample subset is showed in Table 1.

**Figure 3:**
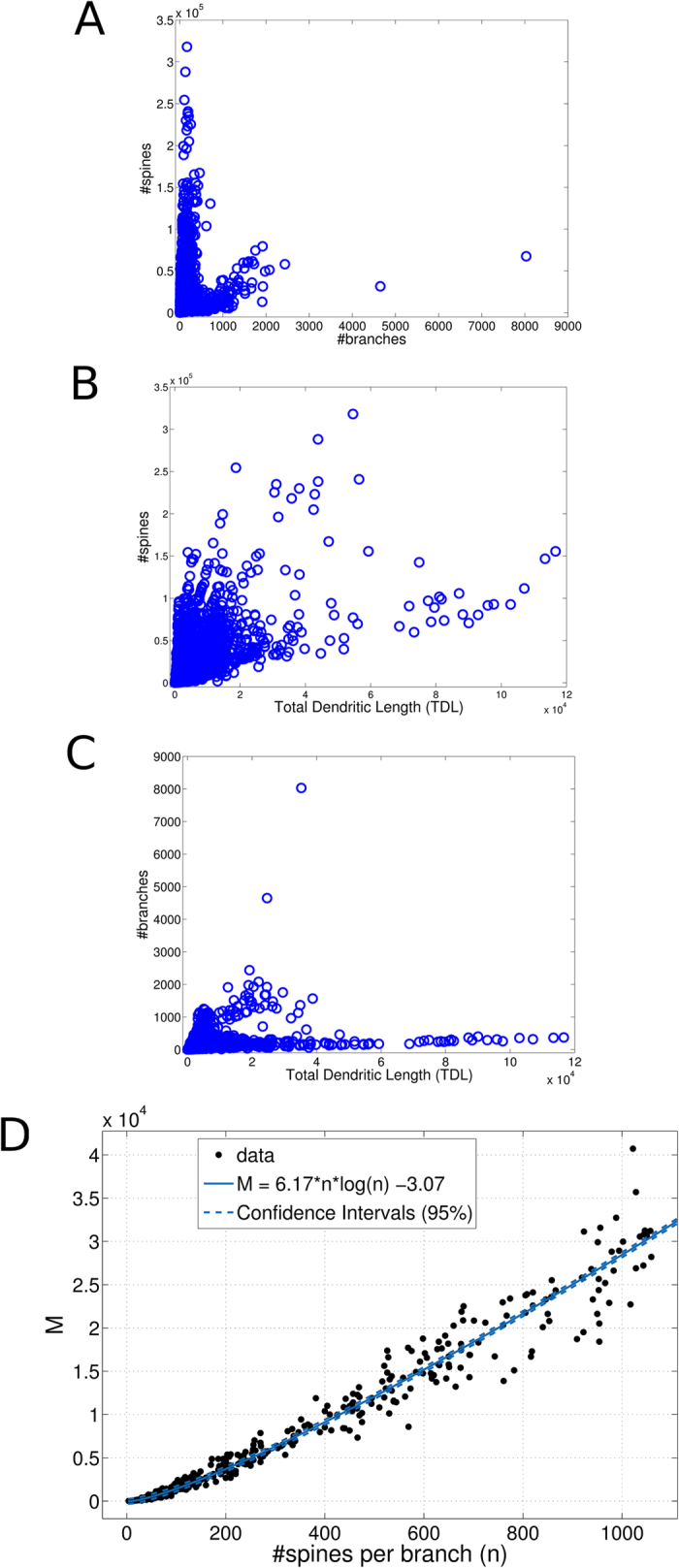
Analysis of dendritic morphological features within the neuron reconstructions of the neuromorpho dataset (v. 5.6) and the rule (M) extraction for the quantification of the discriminable patterns. (**A**) The number of dendritic branches and the number of spines show a weak correlation (*R* = 0.231) implying that a high number of branches prevents an elevated number of spines. (**B**) The relationship between the total dendritic length and the putative number of spines extracted by the equation (4) reveals a conspicuous correlation (*R* = 0.560). (**C**) The relationship between the total dendritic length and the putative number of dendritic branches suggests that most neurons with high total dendritic lengths have few branches (*R* = 0.326). Plots A-C are generated used all repository cells (10004). By uniformly selecting 100 cells, the number *M* of discriminable patterns per each branch of the cells has been estimated and a predicting law has been extracted. (**D**) The inferred relationship between the number of spines per branch and the number of discriminated input patterns *M*, the goodness of fit with the adjusted r-square was 0.996. The fitting was computed with the Matlab Curve Fitting Toolbox.

We early inspected some basilar relationships among the number of dendritic branches, the total dendritic length and the number of spines (Fig. 3A–C) to the entire neuromorpho repository by assuming the spine density implied by the Cuntz’s law. We found weak correlations between the number of branches and the number of spines (Fig. 3A, *R* = 0.231, *p* < 0.007, permutation test) and between the total dendritic length and the number of branches (Fig. 3C, *R* = 0.326, *p* < 0.003, permutation test) but a substantial correlation between the total dendritic length and the number of spines (Fig. 3B, *R* = 0.560, *p* < 0.000, permutation test). These results indicated that at least two morphological features (e.g. the number of branches and the number of spines) are required to capture most of the dendritic morphological information and this drove our searching for an analytical law.

The computational approach adopted in this work did not allow the appraisal of the biophysical properties of each reconstructed neuron; hence we adopted an optimization strategy, based on genetic algorithms, which selected within a parameter space the best parameters that maximized M. By applying this framework to a randomly selected pool of 100 cells, we found that the M values fit very well with a *a*·*n*·log *n* + *b* law (adjusted r-square = 0.996, Fig. 3D) where *n* is the number of spines per branch. One more relationship (*a*·*x*^*2*^ + *b*·*x* + *c*) reached the same goodness of fit but had three parameters thus we preferred the previous simplest one and the linear model *a*·*x* + *b* fitted worse (adjusted r-square = 0.865).

Subsequently, we inferred that the equivalent value of *M* for a neuron was the sum of the relative *M* values for each dendritic branch of such neuron 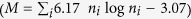. By having a computationally fast equation to accurately estimate the number of discriminable input patterns totally based on the morphological features of the neuron, we explored the consequences of such law along the entire neuromorpho dataset.

**Figure 4:**
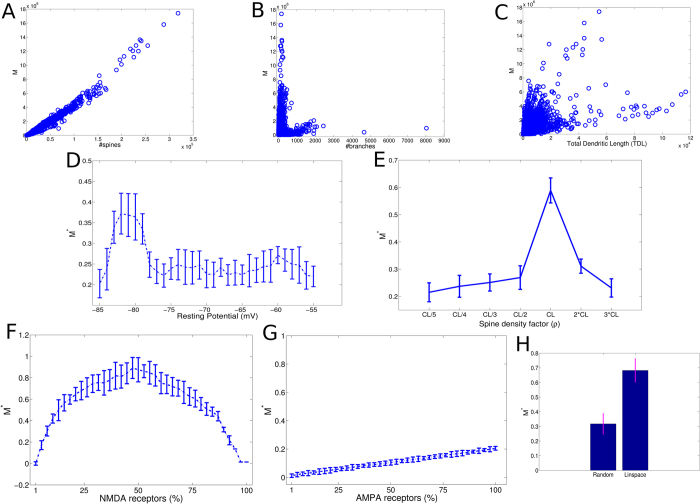
Dependency of M to morphological features and biophysical properties. (**A**) The relationship between the number of spines and *M* shows a strong correlation (*R* = 0.949). (**B**) The relationship between the number of dendritic branches and *M* (*R* = 0.145). (**C**) The relationship between the total dendritic length and *M* (*R* = 0.510). Plots A-C are generated used all repository cells (10004). (**D**) The relationship of M to the resting potential indicates significant increment of M in the hyperpolarized range [−83,−79] mV (**E**) The influence of spine density on the M clearly showed that the density predicted by the Cuntz’s law (CL) produced the best input discriminability (except for primate neocortical neurons where CL/2 was better). (**F**) The effects on M when the percentage of NMDA receptors varied and each spine had an AMPA receptor. (**G**) The effects on M when the percentage of AMPA receptors varied and each spine had an NMDA receptor. (**H**) The spatial distribution of the spine along dendritic segments substantially affected M showing a strong preference for a deterministic scheme where the interdistance among spines was constant.

Hence we analyzed the behavior of M in comparison to the other morphological features and we found that it was tightly correlated with the number of spines (Fig. 4A, *R* = 0.982, *p* < 0.000, permutation test) and with the total dendritic length (Fig. 4C, *R* = 0.617, *p* < 0.000, permutation test) and weakly related with the number of branches (Fig. 4B, *R* = 0.145, *p* < 0.000, permutation test). From these results, we concluded that neurons having highest values of M had relatively few branches and that highest numbers of dendrites (>1000) preclude high M values. In the last analysis of this session, we further investigated the dependency of M on each biophysical property. In this part, we standardized M values in the range [0,1] (feature scaling, M*) in order to make comparable neurons with very different lengths and morphologies. We found that the resting potential appeared very influent on M because, in the range [−83, −79] mV, the number of discriminable inputs was considerably higher (*p* < 0.000, Kruskal-Wallis test, Fig. 4D). Essentially, hyperpolarized cells recognized more input patterns. In addition, we found that the synaptic density suggested by the Cuntz’s law (CL) was the density that produced the best input discriminability (*p* < 0.000, Kruskal-Wallis test, Fig. 4E). In particular, the CL density maximized M for neurons of many types and species except for the neocortical neurons of primates where CL/2 performed better. Further, we analyzed the behavior of M for different concentrations of AMPA and NMDA. The Fig. 4F shows that, when each spine had a AMPA receptor while the number of the randomly assigned NMDA receptors varied, the cells maximized their input discriminability when the NMDA concentration was nearby the 50% of the number of spines (*p* < 0.000, Kruskal-Wallis test). Vice versa, the variation of the AMPA concentration produced weaker effects on M, although significant influences were observed (*p* < 0.007, Kruskal-Wallis test, Fig. 4G), and the maximum of M occurred for the AMPA concentration of 100%. This result suggests that NMDA receptors were more influent than AMPA for the input discriminability and that there exists a specific AMPA/NMDA ratio (2:1) which brought the cell in the best functional regime for the input discriminability. Eventually, we also analyzed two models for the spatial distribution of spine along dendrite segments and we found that the equidistant spine model (Linspace) preferentially maximized M (*p* < 0.000, ranksum test, Fig. 4H). Even in this case, random spine locations were typically preferred in neocortical neurons without however obvious distinction of species, neuron type or cortical layer.

### Neuroanatomical Comparisons

Once established a quantitative interpretation of the dendritic tree in terms of storage capacity, we proceeded by comparing the number of dendritic branches across animal species, brain regions and neuron types. Although the biophysical parameters adopted in the previous analysis have been chosen following a computational perspective that could not have an appropriate biological plausibility, we decided to perform such a comparative analysis speculating on the result consistency and robustness.

Neuron reconstructions were taken from the neuromorpho.org repository which is the largest collection of publicly accessible neuronal reconstructions gathering 10004 neurons of 18 cell types, in 17 brain regions and from 15 animal species (neuromorpho version 5.6, up to May 2014). Across the entire collection, neurons had an average M value of 501549 (SD = 857135, the root of the phylogenetic tree in Fig. 3B) with important variances in the diverse classifications. So in general, by the electrodynamical mechanisms inserted in the neuron reconstruction models, a single neuron can distinguish more than half million of correlated inputs dispersed in their dendritic branches.

### Species

**Figure 5:**
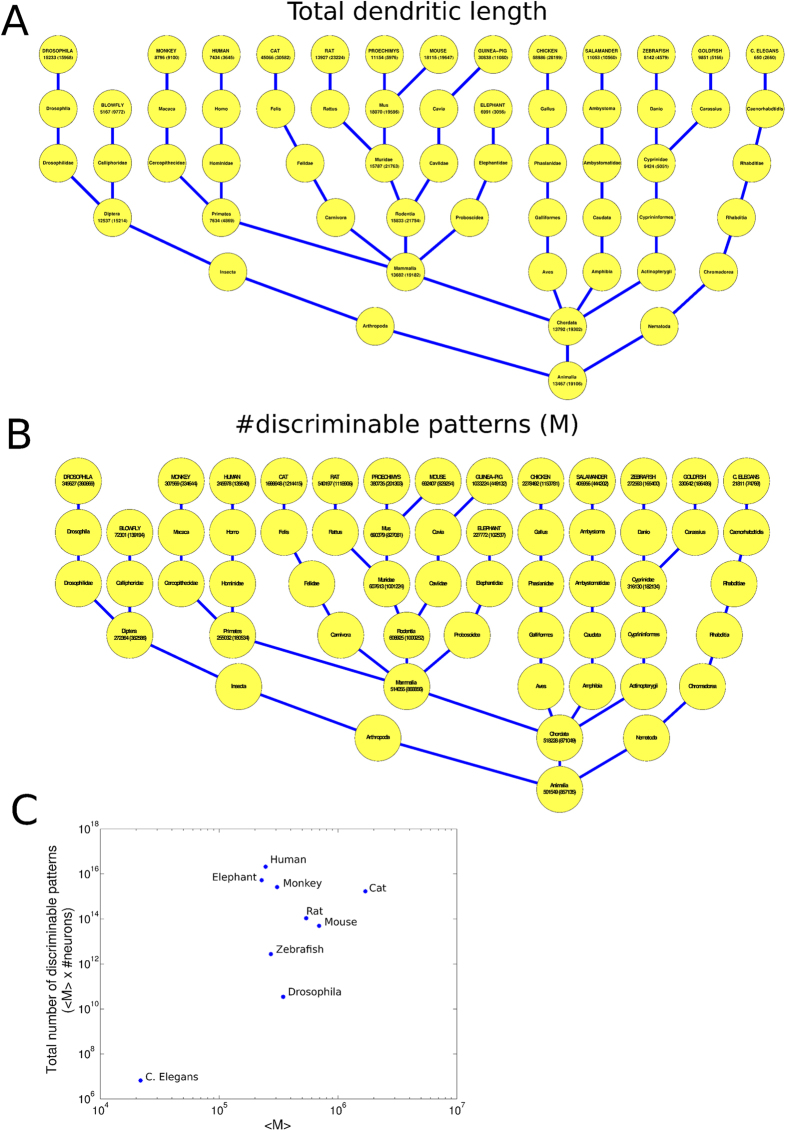
Phylogenetic characterizations of the main species that populate the neuromorpho.org repository. Nodes between root and leaves represent the scientific classification respectively in kingdom, phylum, class, order, family and genus (if applicable). The phylogenetic characterization of the average total dendritic length (**A**) and of the average numbers of discriminable input patterns, *M*. (**C**) A sample of species has been weighed by multiplying the total number of central nervous system neurons with the average M values. The elevated number of neurons in primates and elephant place them in the first position for total number of discriminable patterns. Axes are in logarithmic scales. We considered the following number of neurons: 302 neurons for C. Elegans, 100.000 for Drosophila, 10.000.000 for Zebrafish, 7.1 × 10^7^ for Mouse, 2 × 10^8^ for Rat, 1 × 10^9^ for Cat, 8.5 × 10^9^ for Monkey, 2.3 × 10^10^ for Elephant and 8.5 × 10^10^ for Human. Numbers in brackets indicate the standard deviation.

We first compared dendritic tree morphologies across the animal species and we selected 15 species out of the 20 present in the neuromorpho repository putting aside scarcely represented species (agouti, cricket, rabbit, turtle and lobster with less than 15 reconstructed neurons). The number of samples and the brain regions, which they came from, are reported in Table 2 while Fig. 5A–B shows the phylogenetic trees of the analyzed animal species. Leaves of tree contain capitalized words that illustrate the exact name of species and nodes between the root and leaves represent the scientific classification respectively in kingdom, phylum, class, order, family and genus (if applicable). Numbers below names report features (the total dendritic length in Fig. 5A or M in Fig. 5B) of neurons of that species (second numbers indicates standard deviations). All pairwise comparisons below were performed with the non-parametric Wilcoxon ranksum test and p-values were smaller than 0.000 except when diversely specified.

An early phylum classification showed that neurons from Chordata had M values ~90% higher than Arthropoda and 2275% higher than Nematoda. Interestingly, Rodentia had higher M values (+138%) than Primates. Also Cyprinidae (+24%) and Ambystomatidae (+61%) had higher M values in comparison to Primates. In particular, humans’ M values only overcame C. elegans (+1027%) and blowflies (+241%) and were statistically equivalent to monkeys (P = 0.172), drosophila (P = 0.525) and elephants (P = 0.317). Such results were quite unexpected because primate and elephant brains are classified as more developed^23^ in terms of cognitive ability, awareness, etc. We therefore tried to weight the previous rank by multiplying the average M value of each species with the number of total neurons of their own central nervous system (where available). The Fig. 3C illustrates the new scenario where despite the low M values, the human central nervous system gained the first position scoring more than 10^1^6 recognizable patterns followed by elephant, monkey, cat, rat, mouse, zebrafish, drosophila and C. elegans.

By analyzing basilar morphological features neurons of Primates also have less dendritic arborizations (−54%) than Rodentia and have less arborizations (−77%) than Diptera. Remarkably, human neurons have a comparable number of dendritic branches with goldfish (+0.03%, P = 0.187), monkey (−26%, P = 0.074), elephant (−13%, P = 0.240) and more branches than C. elegans (+730%) and crickets (+273%).

Lastly, we questioned if the reduced discrimination capability found in human neurons could hold when we considered only neocortical neurons instead of the entire nervous systems. Again, human neocortical neurons had an average M value of 245978 (SD = 135640) while rodents had the M average set to 487140 (SD = 800201) confirming the general lower capacity of human neurons in comparison to rodents to discriminate dendritic input patterns.

These comparisons highlighted the surprising low rank of human neurons among the analyzed animal species suggesting that the innumerable better cognitive abilities of humans could not be related to the richness of the dendritic storage mechanisms. In conclusion, the results of this section indicate specific evolutionary strategies adopted in primates to augment their memory (i.e. the ability to distinguish patterns), which result in increasing the number of neurons with concurrent reduction of the single neuron memory capacity.

### Neuron Types

In the subsequent comparative analysis, we investigated the discriminability capacity of different types of neuron attainable in the neuromorpho repository. The Table 3 reports the average number of dendritic branches for each inspected type. From a preliminary exploratory analysis, the enormous morphological differences can be appreciated among the neuron types where for instance, motoneuron and Purkinje cells exhibited very intricate and broad dendritic arborizations while von Economo and large aspiny cells featured plain dendritic structures. Since the discriminability M strongly depended on the distribution of the total dendritic length over the tree branches, we expected that neurons with large but poorly branched dendrites should have high M values.

By computing M for each cell types with at least 10 reconstructions (N = 8996), we found that bipolar, multipolar, motoneuron, medium spiny, ganglion and dopamine cells had higher M values (+349%, +258%, +69%, +46%, +42%, +24% respectively) than the average (M = 510148). On the other side, pyramidal (P = 0.389) and stellate (P = 0.108) neurons did not report significant differences in comparison to the general distribution of M values. Moreover, granule (−20%), interneurons (−30%), thalamocortical (−39%), Purkinje (−46%), sensory (−52%), Golgi II (−54%) and I (−67%), large aspiny (−89%), von Economo (−94%) and projection (−95%) cells had lower M values.

In this current ranking, bipolar and multipolar types are represented by few reconstructions both from specific regions: the former from the Nucleus laminaris of the chicken brainstem and the latter from the rat perirhinal cortex. Therefore such results should consider the narrowness of these samples. However, neurons of the Nucleus laminaris are crucially involved in essential sound localization functions especially in birds and reptiles. Furthermore, such neurons are coincidence detectors of sound information and constitute fundamental processing stage of the binaural hearing. Similarly, perirhinal cortices integrate high-level multisensory inputs from many sensory cortices in all mammalian and the high M values could be the result of an increasing evolutionary demand to efficiently distinguish abstract information. Another important cell type which deserves further mentions is the Von Economo neuron which had the lowest M values. Also called spindle neurons, von Economo neurons are implicated in emotions and social behaviors and their reduced capacity to discriminate input patterns could remark their hypothesized role of communicators among high-order cortical areas in large brain animals.

### Brain Regions

In the last comparative analysis we investigated the neuronal discrimination capability of different brain regions. We first divided brain regions in cortical and non-cortical ones and subsequently we selected only those that were abundantly represented (at least two species and more than 10 neurons as sum).

Among the non-cortical regions (see Table 4), spinal cord (+79%), brainstem (+47%), basal ganglia (+27%) and hippocampus (+14%) neurons reported higher M values in comparison to the overall distribution. Instead, amygdala (P = 0.102) and retina (P = 0.847) neurons had M values that showed no statistical significances, while olfactory bulb (−9%), medulla (−54%), ventral thalamus (−60%) and cerebellum (−68%) neurons had significant lower M values on average.

Among the cortical regions (see Table 5), neurons from the primary somatosensory cortex showed higher M values (+54%) while motor cortex (−14%), prefrontal lobe (−29%), occipital lobe (−38%), frontal lobe (−47%) and temporal lobe (−51%) neurons had lesser M values on average in comparison to the overall M distribution.

Lastly, we decided to analyze the M distribution among the most represented human cortical regions (Table 6) and we found that frontal lobe (+12%) and parietal lobe (+11%) neurons had greater M values than other neurons, while prefrontal cortex (P = 0.446) and temporal lobe (P = 0.649) neurons displayed no significant differences. Eventually, occipital lobe (−9%) and anterior cingulate cortex (−65%) neurons had significant lower M values on average. Although most comparisons are significant, relative differences were much less noticeable and M values appeared more uniform than previous distributions.

By integrating all data, we found that non-cortical neurons had much higher M values than cortical neurons and in particular of human cortical regions (+96% and +210% respectively) and therefore that M values of human cortical neurons were smaller (−37%) than those of non-human neurons. Results of this section confirmed the smaller capability of human neurons to discriminate input patterns through synaptic clustering potentially suggesting that network mechanisms of memory allocation were preferred (instead of subcellular ones) in the evolutionary lineage of primates (monkeys and humans) and big mammals (elephants). Further, such a hypothesis can explain the profound discrepancies between the cortical and non-cortical brain regions.

## Discussion

In this paper, we investigated the computational implications of a class of neuronal models which enable autonomous recognition of input patterns within their dendritic branches through differentiated somatic voltage waveforms. We found a predictive rule that remains invariant across a sample of 100 neuron reconstructions of the neuromorpho repository. Indeed, the total number of discriminable patterns by the whole dendritic tree (M) could be approximated by a 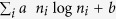 law where *n*_*i*_ was the number of spines along the i^*th*^ dendritic branch and *a* and *b* are two constants. By exploring the entire neuromorpho repository, we found a set of remarkable comparative results spanning animal species, neuron types and brain regions. Interestingly, primates did not exhibit highest number of discriminable patterns per neuron even when considering solely the neocortical neurons, but humans outperformed other species when weighed on the total number of neurons. In addition, non-cortical regions had a minor number of discriminable patterns per neuron in comparison to cortical regions possibly indicating different memory allocating strategies. It could be inferred that non-cortical neurons rely on subcellular mechanisms in contrast to the cortical multicellular/network mechanisms, a distinguishing strategy that potentially may explain primate versus non-primate imbalances.

### Issues in Biophysical Parameter Tunings

The biophysical modeling of neurons requires a plenty of parameters starting from the geometry of the cell to numerous electrochemical specifications of each compartment. The only available knowledge was the cell geometry resulting from neuronal reconstructions. We then chose a set of parameters critical for input discriminability and we fixed the other parameters for all the simulated models. This approach could highlight important implications concerning the biological plausibility of the results. For instance, some neurons do not have a random spatial distribution of the spine while our computational framework might have selected that distribution because it incremented the M estimation. Again, many neurons are not known to operate in the specific resting potential ranges determined by the algorithm, at least in normal physiological conditions.

However, our neuroanatomical comparisons showed a rich repertoire of consistent results which can corroborate the proposed framework. First, synaptic density is considerably higher in rodent brains than in primates^24^ therefore it is reasonable to expect that M may be considerably higher for rodents, as emphasized by our comparisons. In addition, the average dendritic length of rodent neurons is substantially higher than in primates suggesting more important contributes to the input processing^10^,^11^,^12^. Furthermore, cortical motor neurons have giant dendritic trees which finely modulate the impinging complex interplay of central afferents to achieve the balanced output into the corticospinal tracts. A comparable design repeat in the spinal cord motor neurons is also evidenced. This is in agreement with our comparisons which reported for cortical and spinal motoneurons, the highest M values. Finally, it is sound to expect that subcortical regions would have higher M values than cortical regions because the neuronal density and the dendritic lengths are considerably greater in non-cortical structures than in the cortex.

### Information Processing Considerations

Memory and, in general, the ability to store information is an essential evolutionary trait requiring complex associations among spatio-temporal arranged inputs. Such signals, widely heterogeneous, imply the storage of increasing amounts of information. This growing repertoire of inputs conflicts with many biological constraints^25^. In fact, one fundamental limitation is represented by the metabolic cost of neuron signaling which limits the numerosity of neurons.

Therefore, in this contradictory scenario, it became crucial to provide compensatory high memory storage to neurons. In this work, we found that *N* dendritic branches each with *n*_*i*_ spines allow for discriminating more than 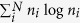 distinct patterns. This can represent a plausible computational breakthrough as such neurons, with several thousand spines along dendritic branches, can recognize hundreds of thousands of different synaptic activation combinations. In addition, from a computational perspective, neurons and neuronal circuits also accomplish the storage demand by compression^26^ suggesting that information can be encoded cheaply. In our experiments, our model performed data reduction of input patterns by encoding large input patterns in voltage waveforms that lasted 100–150 milliseconds. From a theoretical perspective, neurons would maximize its input discriminability (M) by collapsing its dendritic tree into a long single branch. However this simple strategy impoverished the number of active inputs because one of the fundamental roles of dendrites is to provide an adequate spatial covering of the neighboring space that instead is achievable by a tree structure^17^.

### Neuroanatomical Considerations

The importance of the information stored in the different brain regions deserves a dedicated dissertation. Neurons are supposed to differentially represent information at several level of abstraction^27^, hence it is reasonable to assume that some information are more crucial than other. Unexpectedly cortical neurons have less discrimination capability than subcortical neurons. The surprise is justified by the fact that the neocortex processes information of higher order tasks and thus we expected a greater memory power in its neurons. A possible explanation may be searched in the different storage form of information, with different degrees of density, progressively increasing in the scaling up of the neuron rank in a network. Another alternative explanation could be also placed for interpreting the mismatch among the number of discriminable patterns of cortical versus non-cortical neurons. Namely, distributed systems in general and specifically the cortical regions appear to be the highest information distributive systems and, on the other side, show higher resilience to biological insults, that is a superior fault tolerance, coupled to a higher degree of graceful degradation, thus allowing for the instantiation of potential vicarious or compensatory mechanisms. The higher the rank of the brain regions in signal elaboration the higher may be the fault tolerance as individual and species preservation strategy. Losses of high M capacity neurons could be equivalent to less severe functional losses. In addition, from a more extensive perspective, taking into account the whole neuro-glio-vascular compartment, vascular failures can be equiprobable along tissue volumes and because the cortex occupies abundant portions of the total brain volume (77% in humans)^28^,^29^ this strategy would minimize information losses in brain failures.

Another interesting consideration takes into account the metabolic costs related to synaptic transmission: the human brain occupies only the 2% of the body’s mass but it uses more than the 20% of the total energy. In particular, about the 55% of the adenosine triphosphate (ATP) consumed by the brain supplies pre- and post-synaptic mechanisms^30^. In addition, it has been estimated that for a single vesicle release, more than 42% of the energy is drained in NMDA and the 40% by non-NMDA (excluding metabotropic signaling, e.g. mGluR) signaling^31^. Therefore, energy expensive neurons with high memory storages could be metabolically little or not suitable in brains with more than a billion of neurons.

We also propose a further possible interpretation on the surprising discrepancy between cortical neuron higher evolutionary rank and their lower memory capabilities. Greater memory storage abilities in a neuron could be achieved at the expense of fast plasticity and responses in highly loaded networks. The higher the load of a circuitry (as the cortical circuitries convey) the faster the response and adaptivity requirements are expected. A dendritic receptor distribution or branching enabling memory loads could conflict with the need of transience that multiple simultaneous tasks might require. Hence the selective drive toward rapidly adapting neurons in comparison to memory loaded units. The neurodynamic profiles and neurochemistry of the cortex could support this hypothesis. Namely, the strong cortical neuromodulatory component (serotonergic and cholinergic as first) behaves like overall addresser of cortical outputs where the fast components (e.g. the glutamatergic AMPA-NMDA drive at the synapses) could represent the continuously engaged component for fast adaptation to extant conditions. A heavy neuromodulatory component could be conflicting with accumulation storage in neurons where memories should be expressed as they were stored not being affected by the wave of modulators. Complementarily, modulation could instead represent the fast-written-fast-deleted slate where the responses of network low-memory neurons are hosted.

### Limitations and Developments

This work is intended as an exploratory study which inspects the potential opportunities of dendritic morphological features and synaptic clustering in a computational fashion. Many experiments and improvements are required to conclusively settle the results of this work. First of all, the predictive rule for M has been extracted only for 1% of the total available repository because computational times were deeply constraining being nonlinearly proportional to the total dendritic length. Second, although many comparisons were statistically significant, the distribution of neuron reconstruction samples among species, cell types and brain regions was strongly non-uniform. We believe that new versions of the dataset will improve and correct statistics and results (neuromorpho.org has recently released a new version, 5.7, of the repository with more than 3000 additional reconstructions).

At last, although about the 80% of the neuronal activations are glutamatergic, other important neurotransmitters (GABA, acetylcholine, dopamine, serotonin, etc.) could play an important role in the input discriminability.

## Materials and Methods

One of the aims of this work was to figure out how dendritic morphological features impacted the capability of neurons to discriminate coincident input patterns by taking into account the input grouping mechanism of synaptic clustering along single branches. We used a computational framework that combines Matlab routines with external calls to the NEURON simulator (Fig. 1)^32^.

### Simulating by NEURON and the TREES toolbox

NEURON simulator (version 7.3)^32^ is a widely used tool for biophysical simulations of neurons and networks of neurons. In this work we used a NEURON model based on the biophysical and synaptical properties of the model of Branco *et al* (http://senselab.med.yale.edu/ModelDb/showmodel.asp?model=140828) with active and passive dendritic conductances^8^. Such biophysical and synaptical features are enabled in different reconstructed neurons in order to evaluate different hypotheses. Synapses are driven by two principal glutamate ionotropic receptors: the AMPA (*α*-Amino-3-hydroxy-5-methyl-4-isoxazolepropionic acid) and the NMDA (N-Methyl-D-aspartic acid) receptors.

Neuron reconstructions are previously downloaded into local directories and loaded through a modified version of the load_tree function of the Matlab TREES toolbox^17^. Subsequently, the cell geometry file is generated by the neuron_tree function (a modified version allows better interoperability with the NEURON environment) while other TREES toolbox functions collected morphological statistics (len_tree.m, vol_tree.m, dissect_tree.m). Furthermore, a couple of files specified biophysical behaviors of membranes, channels and synapses were so loaded into the NEURON environment attaching to the cell morphology, active and passive dendritic conductance and AMPA and NMDA receptors in the synaptic points. The source code of the entire computational framework can be downloaded at https://sites.google.com/site/antoniogiulianozippo/codes.

### Discriminability of Somatic Waveforms

One of the working hypothesis of this work was that dendrites have the capability to provoke unequivocal voltage somatic waveforms to clusters of synaptic activations along a single branch of the dendritic tree. Since, the central aim was to quantify the discriminability of the somatic waveforms, we designed a formal notion of waveform discriminability and we developed an algorithm to efficiently estimate it. Let be 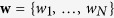 and 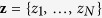 the sequence of data points of two equal-length waveforms (i.e. post-synaptic potentials, action potentials or both). If there exists a set 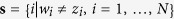, with 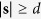, i.e. **w** and **z** are dissimilar in at least *d* points (*d* serves as distance parameter), then **w** and **z** are said *discriminable*, otherwise *non discriminable*.

We collected somatic waveforms from NEURON simulations that had a static representation of 2 integer digits and 6 floating digits expressing millivolts. Typical patch-clamp electrophysiological recording setups are accompanied by noise levels of 10–20 *μ*V and for this reason we truncated our collected voltage waveforms to the second floating digit obtaining the equivalent precision of 10 *μ*V. In addition, the somatic waveform recordings lasted 200 ms with a dt set to 25 *μ*s gathering 8000 voltage data points for each waveforms (*N* = 8000). The threshold *d* was set to 400 (equivalent to 10 ms) and higher values of *d* tended to discriminate less waveforms, vice versa, smaller *d* induced more discriminated waveforms.

At last, we defined a fast algorithm to evaluate large sets of waveforms that returns the number of discriminable ones. It builds a distance matrix, later used to isolate the groups of similar waveforms. The algorithm exploits the disjoint set data structures and the union heuristic to identify the representative waveforms as the produced number of disjoint complete graphs^33^.




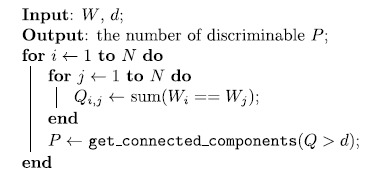




**Algorithm 1:** Algorithm for the estimation of the number of discriminable waveforms.where 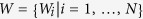 is the set of *N* waveform, 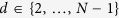 is the threshold for the discriminability (higher values return more discriminable waveforms) and 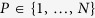 is the estimated number of discriminable waveforms. Binary operators (==, >) applied to a matrix, returns a boolean values matrix and the function get_connected_components(*X*) returns the number of connected components of the graph *G*, represented by the adjacency matrix *X*.

The Fig. 2 shows a toy example with 208 somatic waveforms respectively generated by 208 random activation sequences. As visually appreciable in Fig. 2A, the cell essentially exhibited three shapes (red, purple and yellow). In the first phase the algorithm computes the similarity matrix for each waveform couples (Fig. 2B). By interpreting the obtained matrix as a graph, subsequently the algorithm computes the number of connected components (Fig. 2C) which always coincides with the number of distinguishable waveforms.

The implementation of the Algorithm 0 has been done in Matlab using the CUDA computational framework which speed-up the execution time up to hundredfold (60x on average).

### Estimating Synaptic Distribution over Dendritic Trees

The density and the spatial distributions of axodendritic synapses are generally unknown. In a recent prominent work, Cuntz *et al* proposed and partially validated a simple rule which regulates the total length of a dendritic tree, the number of synapses and the dendrite volume^18^:
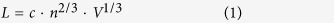
where *L* is the total wiring length, *c* is a proportionality constant, *n* the number of synapses and *V* the total volume. By assuming that each synapsis has a spherical basin of influence, the equation became:
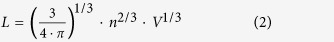
Since we had to know the number of putative spines that an entire dendrite should has, we solved the previous equation in *n* obtaining:
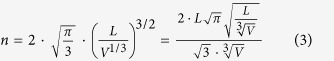


Thus we can calculate the number of putative synapses adduced by dendritic morphology and we derived an equation to distribute spines in dendrites and branches.
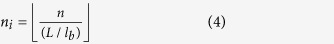
where *l*_*b*_ is the length of branch and *n*_*i*_ is the number of spines in the i^*th*^ branch.

Although the conceived perspective found notable similarities with the available literature, for instance the mouse cortical synaptic density range from 0.5 to 2.1 spines per *μ*m^29^,^34^ and our approach predicted a mean of 1.54 and standard deviation of 0.7, we considered synaptic density values higher and lower than those predicted by Cuntz equation to evaluate possible conditionings on results.

By having *n*_*i*_ for each segment of the dendrite compartments and for each reconstructed neuron, we first performed a theoretical combinatorial consideration about the possible number of combinations of correlated synaptic activations. In general, given *n*_*i*_ the number of available spines and *k* the number of activated spines, the number of possible combinations is computed by the binomial coefficient 
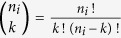
. Therefore, to estimate the possible number of input combinations on a dendritic segment with *l*_*b*_ spines, we obtained:
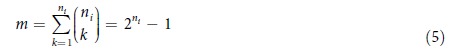


Hence a preliminary theoretical examination proposed an exponential law for the number of possible input activations. Such a relationship produced unfeasible instances even with few tens of spines, therefore discarding the exhaustive search of all possible activation patterns we had to devise an alternative strategy which can be accommodated with current computational architectures. For this reason, we developed a stochastic optimization algorithm to face the intractable number of possible input patterns.

### Stochastic Estimation of the Number of Discriminable Patterns

Our strategy holds on the assumption that when an instance of our NEURON model is exerted with *n* different activation patterns and it recognizes *m* ≤ *n* of them, then when the same model is exerted with a number *N* > *n* of patterns, it should recognize a number *M* ≥ *m* of them otherwise the model already expressed the maximum number (*m*) of discriminable patterns. Essentially, we assumed the function of the number of discriminable patterns was a strictly growing function. Taking into consideration this assumption, we elaborated a stochastic estimation strategy where we looked for a plateau of the function which corresponded to the maximum of the function values. Specifically, the algorithm starts by probing the initial discriminability of the dendritic branch for two incremental number of randomly generated activation patterns and if the discrete derivative of the two values is positive the algorithm goes on otherwise whether the derivative is equal or smaller than zeros it stops and returns the maximum values available at that time. The pseudocode below illustrates the basic computational steps of the presented model:




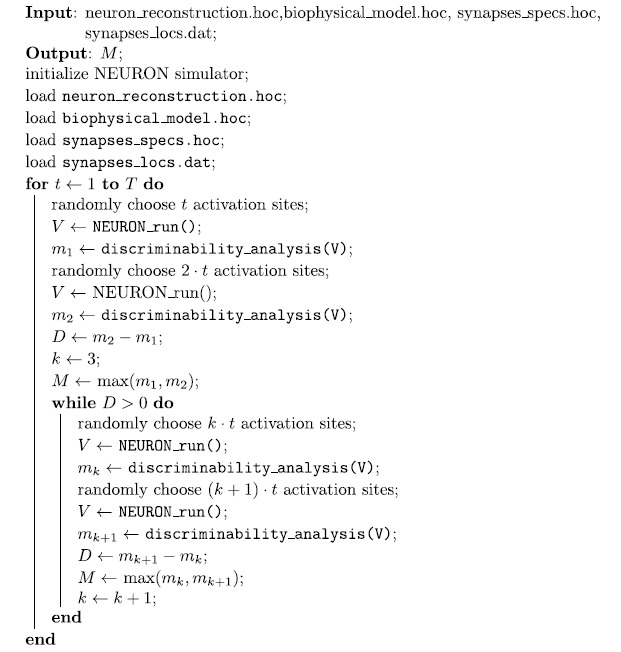




**Algorithm 2:** Algorithm to estimate the maximum number of discriminable patterns by a dendritic segment.

The files passed as arguments correspond to list of files needs to NEURON simulations (Fig. 1F) and they are: the specification of the neuron morphology (neuron_reconstruction.hoc), the specification of the biophysical compartment properties (biophysical_model.hoc), the specification of the synaptic properties (synapses_specs.hoc) and the synaptic locations (synapses_locs.dat). The routine returns only the estimated number *M* of discriminable patterns by the dendritic segment. *T* is the putative number of spines computed by equation (4). The function NEURON_run() triggers the execution of the simulation of the NEURON model for 200 ms (dt = 25 *μ*s, synaptic release at the 50 ms) and returns a set of somatic waveforms each of them related to a random synaptic activation sequence. The function discriminability_analysis(V) returns the number of discriminable patterns according to the criteria presented in section and implemented in the Algorithm 1. Finally, *D* represents the current derivative estimation, the stop criterion of the while loop.

The functioning of the algorithm can be better illustrated with the help of a toy example. We suppose that we have to estimate the number of discriminable waveforms of a given branch with 40 spines elicited by 7 different activation points (theoretically, there exists 
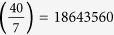
 of possible combinations!), the algorithm first generates 10 random activation sequences and estimate the current value of M (let say 5). Subsequently, it repeats the last step with 20 random activation sequences and it returns a second estimation of M (let say 7). Since, the difference between the two M estimations is positive (*D* = 7 − 5 = 2) the greedy strategy imposes to run further searching for higher values of M. Thus, the algorithm proceeds with 30 random sequences and so forth until the current estimation of M is lower (or equal) than the last one. At this point, the algorithm ends returning the highest observed values of M.

The Fig. 2D–G shows the estimation of M for the cell *Cell-1a* (displayed in Fig. 2E) from the neuromorpho repository^35^,^36^. In particular, the discriminable somatic EPSPs for branches number 19 and 1 are showed respectively in Fig. 2D,F.

### Tuning of Synaptic and Biophysical Parameters

The framework developed for the estimation of the number of discriminable input patterns includes several parameters that could significantly influence the results, therefore we devised an optimization analysis that enlightened which model parameters could lead the M estimation. Among the numerous model parameters we selected 5 crucial: the resting potential of the cell (*V*_*rest*_), the dendritic spine spatial distribution, the spine density and the percentages of AMPA and NMDA receptors. The goal of this analysis was to maximize the objective function M by opportunely choosing the 5 parameters. Since the single estimation of M was computational expensive *per se*, we decided to restrict the five-dimensional parameter space in this way:

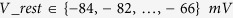
2. Spine Spatial Distribution: 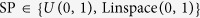; where *U*(0,1) represented the uniformly random distribution while the Linspace(0,1) the equidistant distribution of the spines.
3. Spine Density: 
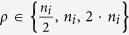
; where *n*_*i*_ is the spine density of the *i*^*th*^ branch derived by the equation (4).
4. Number of AMPA receptors: 
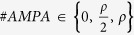
; when the number is diverse by *ρ*, receptors are randomly allocated otherwise each spine has a AMPA receptor.
5. Number of NMDA receptors: 
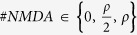
; when the number is diverse by *ρ*, receptors are randomly allocated otherwise each spine has a NMDA receptor.

A simple genetic algorithm selected within the five dimensional parameter space the best choice for a given neuron reconstruction:




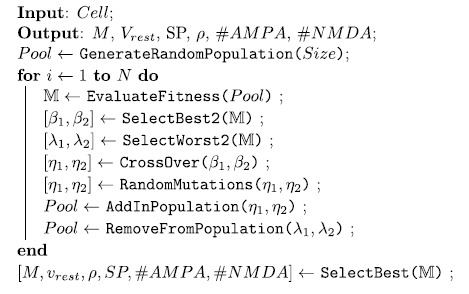




**Algorithm 3:** Algorithm for the selection of the biophysical parameters which maximize M.

The pool size of solutions (*Size*) was initially set to 100 and keeping constant along runs as well as the number of iterations (*N*) fixed to 500. At each step the algorithm obtains the estimations of M for each candidate solution within the *Pool*. The general scheme of the algorithm is composed by three steps: the first selects the best two solutions (the two highest M estimations) by the SelectBest2 function; the second step (CrossOver) randomly swaps the values of the previously chosen solutions; the last step (RandomMutations) imposes with a low probability (0.1 for each of 5 parameters) random modifications to the two new candidate solutions. The functions SelectWorst2, AddInPopulation and RemoveFromPopulation serve to keep constant the pool size. The last step calls the function SelectBest which return the 5 parameters which maximize M.

### Statistical Tests

The significance of correlation coefficients is asserted by a permutation tests. Given two data sequences, we asserted how many times out of 10000 trials, randomly shuffling the element sequence positions we obtained a correlation coefficient greater than 0.05. If the ratio of trials that pass the previous condition was lesser than 0.05 we rejected the null hypothesis otherwise we accepted it.

Statistical comparisons among samples are computed with the non-parametric Wilcoxon signed-rank test with a significance level of 0.05. To compare different distributions of M which take values in distinctive sets, we normalized M values (M*) mapping them into the set {0,1} by using the feature scaling technique^37^.

## Additional Information

**How to cite this article**: Zippo, A. G. and Biella, G. E. M. Quantifying the Number of Discriminable Coincident Dendritic Input Patterns through Dendritic Tree Morphology. *Sci. Rep* **5**, 11543; doi: 10.1038/srep11543 (2015).

## Figures and Tables

**Table 1:** Sample of the analyzed 100 reconstructed neurons used to extract the M law.

Specie/Cell Type/Region	Cell	v_rest_	SP	*ρ*	AMPA	NMDA	M
Rat/Dopamine/Basal Ganglia	Nigra11h941-1	−80	LS	CL	NS	NS/2	133564
Mouse,Thalamocortical,Ventral Thalamus	Cell-1a	−82	LS	CL	NS	NS/2	23369
Rat/Stellate/S1	AK137sdaxlay	−80	LS	CL	NS	NS/2	57411
Chicken/Bipolar/Brainstem	10-2911-XDCT-s2-cell1	−78	LS	CL	NS	NS/2	9572
Human/Pyramidal/Parietal Lobe	51-6-7	−80	UN	CL/2	NS	NS/2	2541
Rat/Purkinje/Cerebellum	p20	−82	LS	CL	NS	NS/2	9720
Turtle/Motoneuron/Spinal Cord	5Tmn2	−80	LS	CL	NS	NS/2	337998
Zebrafish/Ganglion/Retina	20061022z166r2c1	−80	LS	CL	NS	NS/2	52297
Monkey/Interneurons/Prefrontal Lobe	03-22-01-5localArbor	−80	LS	CL/2	NS	NS/2	47309
Rat/Granule/Hippocampus	No40-B-TTX	−80	LS	CL	NS	NS/2	6649
Elephant/Pyramidal/Neocortical Layer 2/3	155-1-5k	−80	LS	CL/2	NS	NS/2	80219
Blowfly/Interneuron/Visual Lobe	HSE-fluoro02	−80	LS	CL	NS	NS/2	6628

The 5 biophysical parameters of each neuron have been selected by a genetic algorithm that maximizes M. The first column indicates the specie, the cell type and the nervous system region from which the cell was extracted. The second column reports the name used in the neuromorpho.org repository. The third column contains the membrane resting potential (*V*_*rest*_, mV). The fourth column represents the spine spatial distribution (SP) which could be the Linspace (LS) or the uniform distribution (UN). The fifth column shows the spine density where CL indicates the value suggested by the Cuntz’s law. The sixth and seventh columns report the number of AMPA and NMDA receptors allocated along the branches. NS stands for number of spines meaning that each spine had the receptor, otherwise NS/2 indicates that only half of spines had the specific receptor. The last column represents the maximum value of M obtained for the cell.

**Table 2:** Features of neurons extracted from the selected 15 animal species.

Species	N	N. dendritic branches	Nervous System Sites
Blowfly	56	741.12 ± 1197.35	Visual Lobe
C. Elegans	302	7.74 ± 36.62	Entire system
Cat	103	216.37 ± 168.23	Primary Visual Cortex, Motor Cortex, Brainsteam, Thalamus
Chicken	34	184.18 ± 54.06	Brainsteam
Drosophila	398	277.63 ± 452.14	Peripheral, Olfactory Bulb, Protocerebrum
Elephant	76	64.10 ± 36.44	Neocortex, Occipital Lobe, Frontal Lobe
Goldfish	100	54.81 ± 31.68	Retina, Retinal Ganglion Cells, Optic Nerve
Guinea Pig	19	302.32 ± 257.96	Cerebellum, Hippocampus
Human	2147	56.71 ± 16.42	Frontal Lobe, Temporal Lobe, Parietal Lobe, Occipital Lobe, Prefrontal Lobe
Monkey	376	77.60 ± 116.17	Prefontal Cortex, Primary Visual Cortex, Temporal Lobe
Mouse	2726	120.95 ± 169.36	Amygdala, Hippocampus, Temporal Lobe, Frontal Lobe, Visual Cortex, Somatosensory Cortex, Prefrontal Cortex, Entorhinal Cortex, Spinal Cord, Basal Ganglia, Olfactory Bulb, Retina Ganglion Cells, Cerebellum, Hypothalamus, Thalamus, Medulla, Peripheral
Proechimys	17	69.22 ± 37.70	Hippocampus
Rat	3337	138.58 ± 244.67	Amygdala, Hippocampus, Temporal Lobe, Frontal Lobe, Visual Cortex, Somatosensory Cortex, Prefrontal Cortex, Motor Cortex, Entorhinal Cortex, Spinal Cord, Basal Ganglia, Anterior Olfactory Nucleus, Retina Ganglion Cells, Cerebellum, Hypothalamus, Thalamus, Medulla, Basal Forebrain
Salamander	64	62.54 ± 40.37	Retinal Ganglion Cells
Zebrafish	26	44.59 ± 25.91	Retinal Ganglion Cells, Spinal Cord
**Overall**	9781	115.23 ± 223.5	—

The second column indicates the number of cells used from that specie, the third column indicates the average number of dendritic branches (the second number is the standard deviation). The last column represents the brain regions where the selected cells are extracted.

**Table 3:** Features of neurons extracted from 18 neuron types.

Neuron type	Pro.venience	N	#branches	TDL (*μ*m)	M
Purkinje	Mouse, Rat	10	821.20 ± 146.47	6929 ± 2233	275362 ± 132460
Sensory	C. Elegans, Drosophila	209	461.09 ± 564.69	5132 ± 7139	248993 ± 362051
Interneurons	Blowfly, C. Elegans, Cat, Mouse, Rat	1452	255.20 ± 402.12	3045 ± 3029	353655 ± 534992
Bipolar	Chicken	32	193.87 ± 34.88	1177 ± 303	2293912 ± 1072838
Multipolar	Rat	15	173.86 ± 37.61	11166 ± 1462	1829897 ± 516440
Stellate	Mouse, Rat	57	144.70 ± 266.16	4957 ± 3336	466546 ± 646603
Ganglion	Goldfish, Mouse, Rat, Salamander, Zebrafish	959	125.95 ± 92.57	3745 ± 1769	724465 ± 686886
Dopamine	Rat	42	98.57 ± 135.76	4325 ± 2882	635270 ± 683151
Pyramidal	Cat, Elephant, Guinea Pig, Human, Monkey, Mouse, Rat	4923	84.23 ± 140.67	4357 ± 3789	504499 ± 994107
Motoneuron	C. Elegans, Cat, Mouse, Rat, Zebrafish	215	84.04 ± 165.75	16916 ± 27747	866164 ± 1346482
Thalamocortical	Cat, Mouse	33	78.51 ± 96.18	4481 ± 7092	312314 ± 572314
Projection	Rat	30	52.06 ± 20.99	1762 ± 545	26487 ± 10459
Granule	Mouse, Rat	341	39.26 ± 32.52	2049 ± 1339	407316 ± 595576
Medium spiny	Mouse, Rat	427	34.88 ± 18.02	1604 ± 684	745074 ± 627819
Golgi Type II	Rat	26	34.50 ± 12.12	1407 ± 389	232128 ± 94758
Golgi Type I	Rat	50	23.28 ± 9.08	919 ± 353	168101 ± 67738
Large aspiny	Rat	146	22.58 ± 10.87	1736 ± 773	54408 ± 35531
Von Economo	Human	29	12.01 ± 7.70	1040 ± 475	29040 ± 17782
**Overall**	—	8996	82.35 ± 164.68	4085 ± 5751	510148 ± 874737

The second column represents the brain regions where the selected cells are extracted, the third indicates the number of cells, the fourth column indicates the average number of dendritic branches. The fifth column shows the total dendritic length expressed in *μ*m. The last column represents the average values of *M*. Numbers after ± express the standard deviation.

**Table 4:** Features of neurons extracted from 10 brain non-cortical regions.

Non cortical regions	N	Species	#branches	TDL (*μ*m)	M
Amygdala	47	Mouse, Rat	68.70 ± 27.45	4790 ± 2115	344599 ± 360923
Basal Ganglia	243	Mouse, Rat	51.59 ± 38.39	2299 ± 1380	1008280 ± 563147
Brainstem	82	Cat, Chicken, Rat	112.15 ± 91.25	3798 ± 2853	1169589 ± 1214898
Cerebellum	24	Guinea Pig, Mouse, Rat	418.41 ± 365.44	4450 ± 2755	250204 ± 105706
Hippocampus	1151	Guinea Pig, Mouse, Proechimys, Rat	141.62 ± 287.27	5386 ± 6556	902778 ± 1684057
Medulla	131	Mouse, Rat	180.97 ± 182.99	1108 ± 558	357693 ± 321478
Olfactory bulb	296	Drosophila, Mouse, Rat	102.46 ± 275.98	1582 ± 1586	72385 ± 85684
Retina	960	Goldfish, Mouse, Rat, Salamander, Zebrafish	111.38 ± 104.42	3493 ± 1948	472425 ± 511419
Spinal Cord	136	Cat, Mouse, Rat, Zebrafish	145.97 ± 132.75	29791 ± 30806	1422872 ± 1497496
Ventral Thalamus	46	Cat, Mouse, Rat	69.89 ± 83.05	4645 ± 6048	316707 ± 486536
**Overall**	3116	—	124.54 ± 228.56	5639 ± 10347	793473 ± 1316950

The second column represents the number of cells, the third indicates the species where the selected cells are extracted, the fourth column indicates the average number of dendritic branches. The fifth column shows the total dendritic length expressed in *μ*m. The last column represents the average values of *M*. Numbers after ± express the standard deviation.

**Table 5:** Features of neurons extracted from 6 brain cortical regions.

Cortical regions	N	Species	#branches	TDL (*μ*m)	M
Frontal Lobe	475	Elephant, Human, Mouse, Rat	119.79 ± 184.59	3984 ± 1506	214012 ± 154991
Motor Cortex	118	Mouse, Rat	50.58 ± 30.71	2759 ± 1599	346175 ± 173221
Occipital Lobe	298	Elephant, Human, Mouse	67.74 ± 63.23	3697 ± 1383	251896 ± 173249
Prefrontal Lobe	890	Human, Monkey, Mouse, Rat	83.20 ± 125.35	3437 ± 2079	287593 ± 413518
Somatosensory Cortex	1368	Agouti, Mouse, Rat	159.13 ± 214.08	4318 ± 3468	623133 ± 938742
Temporal Lobe	141	Human, Mouse	50.86 ± 18.76	2690 ± 1489	197350 ± 126248
**Overall**	3287	—	116.06 ± 173.82	3834 ± 2631	404197 ± 660978

The second column represents the number of cells, the third indicates the species where the selected cells are extracted and the fourth column indicates the average number of dendritic branches. The fifth column shows the total dendritic length expressed in *μ*m. The last column represents the average values of *M*. Numbers after ± express the standard deviation.

**Table 6:** Features of neurons extracted from 6 human brain cortical regions.

Human Cortical Regions	N	#branches	TDL (*μ*m)	M
Anterior Cingulate (ACC)	25	28.48 ± 15.46	2266 ± 1687	89627 ± 83532
Frontal Lobe	200	58.57 ± 18.06	4097 ± 1291	286856 ± 123024
Occipital Lobe	281	58.41 ± 15.48	3561 ± 1061	233081 ± 107225
Parietal Lobe	100	59.24 ± 15.73	4139 ± 1177	284058 ± 98798
Prefrontal Cortex	392	63.25 ± 15.77	4072 ± 1216	263090 ± 115968
Temporal Lobe	100	51.01 ± 14.47	3394 ± 1102	249274 ± 105732
**Overall**	2147	55.25 ± 16.17	3849 ± 1245	256441 ± 116398

The second column represents the number of cells, the third indicates the average number of dendritic branches. The fourth column shows the total dendritic length expressed in *μ*m. The last column represents the average values of *M*. Numbers after ± express the standard deviation.

## References

[b1] Brown C. E., Boyd J. D., Murphy T. H. Longitudinal *in vivo* imaging reveals balanced and branch-specific remodeling of mature cortical pyramidal dendritic arbors after stroke. J Cereb Blood Flow Metab 30, 783–791 (2010). 19920846 10.1038/jcbfm.2009.241 PMC2949167

[b2] Caroni P., Donato F., Muller D. Structural plasticity upon learning: regulation and functions. Nat Rev Neurosci 13, 478–490 (2012). 22714019 10.1038/nrn3258

[b3] Yuste R., Bonhoeffer T. Morphological changes in dendritic spines associated with long-term synaptic plasticity. Annu Rev Neurosci 24, 1071–1089 (2001). 11520928 10.1146/annurev.neuro.24.1.1071

[b4] Segal M. Dendritic spines and long-term plasticity. Nat Rev Neurosci 6, 277–284 (2005). 15803159 10.1038/nrn1649

[b5] Tailby C. Activity-dependent maintenance and growth of dendrites in adult cortex. Proc Natl Acad Sci USA 102, 4631–4636 (2005). 15767584 10.1073/pnas.0402747102 PMC555467

[b6] Papoutsi A. Coding and decoding with dendrites. J Physiol Paris 108, 18–27 (2014). 23727338 10.1016/j.jphysparis.2013.05.003

[b7] London M., Hausser M. Dendritic Computation. Annu Rev Neurosci 28, 503–532 (2005). 16033324 10.1146/annurev.neuro.28.061604.135703

[b8] Branco T., Clark B., Hausser M. Dendritic discrimination of temporal input sequences in cortical neurons. Science 329, 1671–1675 (2010). 20705816 10.1126/science.1189664 PMC6354899

[b9] Branco T., Hausser M. The single dendritic branch as a fundamental functional unit in the nervous system. Curr Opin Neurobiol 20, 494–501 (2010). 20800473 10.1016/j.conb.2010.07.009

[b10] Gollo L. L., Kinouchi O., Copelli M. Active dendrites enhance neuronal dynamic range. PLoS Comp Biol 5, 10.1371/journal.pcbi.1000402 (2009). PMC2690843 19521531

[b11] Spruston N. Pyramidal neurons: dendritic structure and synaptic integration. Nat Rev Neurosci 9, 206–221 (2008). 18270515 10.1038/nrn2286

[b12] Volman V. Locally balanced dendritic integration by short-term synaptic plasticity and active dendritic conductances. J Neurophysiol 102, 3234–3250 (2009). 19759328 10.1152/jn.00260.2009 PMC2804429

[b13] Makino H., Malinow R. Compartmentalized versus global synaptic plasticity on dendrites controlled by experience. Neuron 72, 1001–1011 (2011). 22196335 10.1016/j.neuron.2011.09.036 PMC3310180

[b14] Kleindienst T. Activity-Dependent Clustering of Functional Synaptic Input on Developing Hippocampal Dendrites. Neuron 72, 1012–1024 (2011). 22196336 10.1016/j.neuron.2011.10.015

[b15] Takahashi N. Locally synchronized synaptic inputs. Science 335, 353–356 (2012). 22267814 10.1126/science.1210362

[b16] Legenstein R., Maass W. Branch-specific plasticity enables self-organization of nonlinear computation in single neurons. J Neurosci 31, 10787–10802 (2011). 21795531 10.1523/JNEUROSCI.5684-10.2011 PMC6623094

[b17] Cuntz H. One Rule to Grow Them All: A General Theory of Neuronal Branching and Its Practical Application. PLoS Comp Biol 6, 10.1371/journal.pcbi.1000877 (2010). PMC2916857 20700495

[b18] Cuntz H., Mathy A., Hausser M. A scaling law derived from optimal dendritic wiring. Proc Natl Acad Sci USA 109, 11014–11018 (2012). 22715290 10.1073/pnas.1200430109 PMC3390826

[b19] O’Brien J., Unwin N. Organization of spines on the dendrites of Purkinje cells. Proc Natl Acad Sci USA 103, 1575–1580 (2006). 16423897 10.1073/pnas.0507884103 PMC1360541

[b20] Yadav A. Morphologic Evidence for Spatially Clustered Spines in Apical Dendrites of Monkey Neocortical Pyramidal Cells. J Comp Neurol 520, 2888–2902 (2012). 22315181 10.1002/cne.23070 PMC3573331

[b21] Jammalamadaka A. “Statistical analysis of dendritic spine distributions in rat hippocampal cultures. BMC Bioinf 14, 10.1186/1471-2105-14-278 (2013). PMC3871014 24088199

[b22] Morals J. Random Positions of Dendritic Spines in Human Cerebral Cortex. J Neurosci 34, 10078–10084 (2014). 25057209 10.1523/JNEUROSCI.1085-14.2014 PMC4107399

[b23] Hart B. L., Hart L. A., Pinter-Wollman N. Large brains and cognition: Where do elephants fit in? Neurosci & Biobehav Rev 32, 86–98 (2008). 17617460 10.1016/j.neubiorev.2007.05.012

[b24] Ballesteros-Yánez I. Density and morphology of dendritic spines in mouse neocortex. Neuroscience 138, 403–409 (2006). 16457955 10.1016/j.neuroscience.2005.11.038

[b25] Fonseca-Azvedo K., Herculano-Houzel S. Metabolic constraint imposes tradeoff between body size and number of brain neurons in human evolution. Proc Natl Acad Sci USA 109, 18571–18576 (2012). 23090991 10.1073/pnas.1206390109 PMC3494886

[b26] Tetzlaff C. Times scales of memory, learning, and plasticity. Biol Cybern 106, 715–726 (2012). 23160712 10.1007/s00422-012-0529-z

[b27] Quian Quiroga R. Invariant visual representation by single neurons in the human brain. Nature 435, 1102–1107 (2005). 15973409 10.1038/nature03687

[b28] Herculano-Houzel S. The human brain in numbers: a linearly scaled-up primate brain. Front Hum Neurosci 3, 10.3389/neuro.09.031.2009 (2009). PMC2776484 19915731

[b29] DeFelipe J. The evolution of the brain, the human nature of cortical circuits, and intellectual creativity. Front Neuroanat 5, 10.3389/fnana.2011.00029 (2011). PMC3098448 21647212

[b30] Attwell D., Laughlin S. An energy budget for signaling in the grey matter of the brain. J Cereb Blood Flow Metab 21, 1133–1145 (2001). 11598490 10.1097/00004647-200110000-00001

[b31] Harris J., Joliver R., Attwell D. Synaptic Energy Use and Supply. Neuron 75, 762–777 (2012). 22958818 10.1016/j.neuron.2012.08.019

[b32] Hines M. L., Carnevale N. T. NEURON: a tool for neuroscientists. Neuroscientist 7, 123–135 (2001). 11496923 10.1177/107385840100700207

[b33] Knight K. Unification: A Multidisciplinary Survey. ACM Comput Surv 21, 215–225 (1989).

[b34] Schuz A., Palm G. Density of neurons and synapses in the cerebral cortex of the mouse. J Comp Neurol 286, 442–455 (1989). 2778101 10.1002/cne.902860404

[b35] Ascoli A.G. Mobilizing the base of neuroscience data: the case of neuronal morphologies. Nat Rev Neurosci 7, 318–324 (2006). 16552417 10.1038/nrn1885

[b36] Ascoli A.G., Donohue D.E., Halavi M. NeuroMorpho.Org: a central resource for neuronal morphologies. J Neurosci 27, 9245–51 (2007). 10.1523/JNEUROSCI.2055-07.2007 PMC6673130 17728438

[b37] Aksoy S., Haralick R. Feature normalization and likelihood-based similarity measures for image retrieval. Pattern Recogn 22, 563–582 (2011).

